# Combating Parasitic Nematode Infections, Newly Discovered Antinematode Compounds from Marine Epiphytic Bacteria

**DOI:** 10.3390/microorganisms8121963

**Published:** 2020-12-11

**Authors:** Nor Hawani Salikin, Jadranka Nappi, Marwan E. Majzoub, Suhelen Egan

**Affiliations:** 1 Centre for Marine Science and Innovation, School of Biological, Earth and Environmental Sciences, UNSW, Sydney, NSW 2052, Australia; norhawani0203@gmail.com (N.H.S.); j.nappi@unsw.edu.au (J.N.); m.majzoub@unsw.edu.au (M.E.M.); 2 School of Industrial Technology, Universiti Sains Malaysia, USM, 11800 Penang, Malaysia

**Keywords:** antinematode compound, anthelminthic drugs, marine epiphytic bacteria, marine biofilm, marine environment, parasitic nematode, *Caenorhabditis elegans*

## Abstract

Parasitic nematode infections cause debilitating diseases and impede economic productivity. Antinematode chemotherapies are fundamental to modern medicine and are also important for industries including agriculture, aquaculture and animal health. However, the lack of suitable treatments for some diseases and the rise of nematode resistance to many available therapies necessitates the discovery and development of new drugs. Here, marine epiphytic bacteria represent a promising repository of newly discovered antinematode compounds. Epiphytic bacteria are ubiquitous on marine surfaces where they are under constant pressure of grazing by bacterivorous predators (e.g., protozoans and nematodes). Studies have shown that these bacteria have developed defense strategies to prevent grazers by producing toxic bioactive compounds. Although several active metabolites against nematodes have been identified from marine bacteria, drug discovery from marine microorganisms remains underexplored. In this review, we aim to provide further insight into the need and potential for marine epiphytic bacteria to become a new source of antinematode drugs. We discuss current and emerging strategies, including culture-independent high throughput screening and the utilization of *Caenorhabditis elegans* as a model target organism, which will be required to advance antinematode drug discovery and development from marine microbial sources.

## 1. Introduction

Humans are vulnerable to infectious diseases caused by parasitic helminths (nematodes) resulting in morbidity and mortality within the population [1,2,3]. Approximately 24% of the global human population, corresponding to 1.5 billion people, suffer from parasitic helminth infections [4]. There are almost 300 nematodes associated with zoonotic diseases that are able to infect humans, including some of the most devastating parasites such as *Ascaris lumbricoides* (roundworm), *Ancylostoma duodenale* (hookworm), *Gnathostoma spinigerum*, *Halicephalobus gingivalis* and *Trichinella spiralis* (Trichina worm) [1,5,6]. While some parasitic nematodes (e.g., *Ancylostoma duodenale*, *Strongyloides stercoralis* and *Halicephalobus gingivalis*) can penetrate human skin or invade through existing skin lesions [7,8], several parasites infect humans via ingestion of food products contaminated with the embryonated eggs (e.g., *Ascaris lumbricoides* and *Trichuris trichiura*) [9,10] or from eating raw or undercooked freshwater fish, birds, frogs or reptiles contaminated with the parasitic nematode larvae (e.g., *Gnathostoma spinigerum*, *Dracunculus medinensis*, *Eustrongylides* sp. and *Trichinella spiralis*) [11,12,13]. Apart from causing diseases, a high nematode burden also reduces human fecundity [14] and affects children through malnutrition, stunted development and cognitive delay [15,16].

Plant parasitic nematodes (PPNs) also cause diseases [17,18] either by damaging the root system, retarding the plant development or by exposing plants to secondary bacterial, fungal or viral infections [19,20,21,22]. It is estimated that damages caused by PPNs result in a >12% loss in global crop productivity and an average annual loss of ~US$215 billion [23]. Parasitic nematodes also impede the productivity of fisheries and aquaculture industries, resulting in worldwide economic losses and health hazards (i.e., zoonotic diseases or allergies) for consumers [24,25]. For example, the global financial loss in the finfish industry due to parasitic infection is estimated to be as large as US$134 million per annum [24]. Major fish products such as Atlantic mackerel, herring, European hake, Atlantic cod and anchovy are commonly associated with parasitic nematodes e.g., *Anisakis* sp. and *Pseudoterranova* sp. [26], which are transmitted to humans via ingestion of undercooked or raw fish [27,28]. Furthermore, parasitic nematodes cause different pathophysiological symptoms in livestock, reduce meat quality and cause animal mortality [29,30]. In Australia, the economic loss due to parasite infection (e.g., *Ostertagia* sp. and *Trichostrongylus* sp.) and the cost of control management is estimated to be ~AU$1 billion [31,32,33] whereas in Kenya, South Africa and India, ~US$26, 46 and 103 million is spent just to control the *Haemonchus contortus* nematode infection of livestock, respectively [34,35]. While diminishing animal fecundity, nematode parasites such as *Ostertagia ostertagi* (brown stomach worm) and *Dictyocaulus viviparus* (lungworm) reduce dairy production levels (~1.25 L/day/animal or 1.6 L/day/cow, respectively) [36,37].

Currently, the most promising longer-term control strategy is dependent on pharmaceutically derived chemotherapeutic treatments to kill parasitic nematodes and/or mitigate the spread of infection [38,39]. Unfortunately, the emergence of drug resistance among parasitic nematodes, due to prolonged treatment and incorrect drug dosage with an unpredictable infection trend as a result of climate change, is alarming [40,41,42,43,44]. The current prevalence of drug resistance is not only limited to the older classes of antinematode drugs but also those introduced in recent years. For example, monepantel resistance in *T. circumcincta*, *Trichostrongylus colubriformis* and *H. contortus* has been reported in New Zealand and the Netherlands within just five years of launching the drug [45,46,47].

Drug resistance has also been reported for almost all of the currently available anthelmintic drugs and nematicide classes including piperazine, benzimidazoles, levamisole, (pyrantel and morantel), paraherquamide, ivermectin (macrocylic lactones and milbemycins), emodepside (cyclodepsipeptides, PF1022A) and nitazoxanide [47,48,49].

Given the impacts of resistant parasitic nematodes to human and economic growth, novel antinematode chemotherapeutic agents are urgently needed as a preventive control against parasite infestation [4,50]. For decades, microorganisms, particularly bacteria, have served as a precious source for bioactive compounds, some of which have been developed into novel drugs including those with nematicidal activity [51,52]. This review highlights the potential of marine epiphytic bacteria as a new repository for newly discovered antinematode metabolites and the underlying mechanism of antinematode compound production by marine microbial biofilms. Furthermore, the utilization of *C. elegans* as a surrogate organism for antinematode drug development will be reviewed.

## 2. Antinematode Drug Discovery: Transition from Terrestrial to Marine-Derived Microbial Compounds

Terrestrial plants and plant extracts have been documented as an ancient therapeutic treatment against parasitic nematodes [53] and today, extensive studies to isolate plant-derived nematicidal compounds are still ongoing [54,55]. However, the re-discovery rate of bioactive metabolites is high [4], reducing the number of newly discovered compounds in the drug discovery pipeline [56]. Moreover, external factors, e.g., specific planting season, environmental temperature and humidity, may also affect the compounds’ reproducibility by the plant producers [4]. Given those inevitable challenges, microbial-derived anthelmintic compounds offer a promising solution [57].

Extensive exploration of terrestrial microbial compounds for therapeutic drug development was initiated in the 20th century [53]. Since then, more than 50,000 beneficial bioactive metabolites have been successfully identified [58,59] some of which have potent nematotoxicity via distinct modes of action (MOAs) (Table 1). Unfortunately, after almost 50 years of drug screening, few newly discovered compounds have been identified from terrestrial-borne microorganisms [59] hence the requirement for a new source of antinematode drug discovery to combat the rapidly growing nematode resistance. Here, the underexplored marine ecosystem, with highly diverse unidentified macro- and microorganisms, represents a new repository for novel nematicidal drugs [59]. In fact, marine bioactive compounds have been acknowledged as having substantial chemical novelty compared to terrestrial metabolites [60].

The marine environment represents the largest biome on the earth (70% of the earth surface; ~361 million km^2^ with average depth of 3730 m) and provides a habitat for a wide diversity of life that outnumbers terrestrial environments [61,62,63,64]. Microorganisms are abundant in this ecosystem (10^5^ to 10^6^ of cells per milliliter), reaching an average 10^28^ to 10^29^ cells either in the open ocean, deep sea, sediment or on the subsurface [64,65,66]. However, the majority of marine bacteria remain unrecognized or uncultivable [67]. Given the enormous diversity and untapped bioactive potential, marine microorganisms are likely to produce newly discovered bioactive compounds with a well-defined molecular architecture and biological function including nematicidal activities [68,69].

**Table 1:** Examples of nematicidal compounds produced by bacteria isolated from terrestrial environments and their modes of action (MOAs) against the target nematodes.

Microbial Producer	Compound	Mode of Action	Target Nematode	Affected Nematode Region	Reference
*Bacillus thuringiensis*	Crystal toxin Cry5B, Cry21A	Toxin binds to nematode glycoconjugate receptor and disrupt the intestinal cells membrane integrity. This action causes fall of nematode brood size and mortality	*Ancylostoma ceylanicum*, *Ascaris suum*, *C. elegans*	Gastrointestinal system	[70,71,72]
*Bacillus simplex*, *B. subtilis*, *B. weihenstephanensis*, *Microbacterium oxydans*, *Stenotrophomonas maltophilia*, *Streptomyces lateritius* and *Serratia marcescens*	Volatile organic compound (VOC) i.e., benzaldehyde, benzeneacetaldehyde, decanal, 2-nonanone, 2-undecanone, cyclohexene and dimethyl disulfide	VOCs reduce nematode motility and death	*Panagrellus redivivus*, *Bursaphelenchus xylophilus*	Unknown	[73]
*Streptomyces avermectinius*	Avermectin and ivermectin (semi-synthetic)	Compound exposure resulted in pharyngeal paralysis and nematode death	*Haemonchus contortus*, *Brugia malayi*, *C. elegans*	Neuromuscular system	[48,74,75,76]
*Serratia marcescens*	Prodigiosin	Compound is toxic against juveniles larvae and inhibit egg hatching competency	*Radopholus similis*, *Meloidogyne javanica*	Unknown	[77]
*Pseudomonas aeruginosa*	Phenazine toxin (phenazine-1-carboxylic, pyocyanin and 1-hydroxyphenazine)	Phenazine-1-carboxylic shows fast killing activity against nematode in acidic environment whilst pyocyanin is toxic in neutral or basic pH. The toxicity of 1-hydroxyphenazine is not dependent on environmental pH. Continuous exposure to phenazine affects protein homeostasis and causes neurodegeneration	*C. elegans*	Neuromuscular system, cell mitochondria and protein folding	[78,79]
*Pseudomonas aeruginosa*	Exotoxin A and other undetermined effectors	Slow-killing activity against nematode is based on infection-like process thus resulting in accumulation of bacteria in the gut. Continuous exposure leads to ceased pharyngeal pumping, nematode immobility and death	*C. elegans*	Gastrointestinal system	[80]
*Pseudomonas aeruginosa*	Chitinase enzyme	Chitinase degrades nematode cuticle, intestine and egg shell leading to the animal death	*C. elegans*	Cuticle, eggs, gastrointestinal system	[81]
*Pseudomonas plecoglossicida*	Glycolipid biosurfactant	Reduction of nematode development, survival and fecundity	*C. elegans*	Unknown	[82]

## 3. Surface Associated Marine Bacteria: A Reservoir for Novel Antimicrobial and Antinematode Drug Discovery

**Figure 1:**
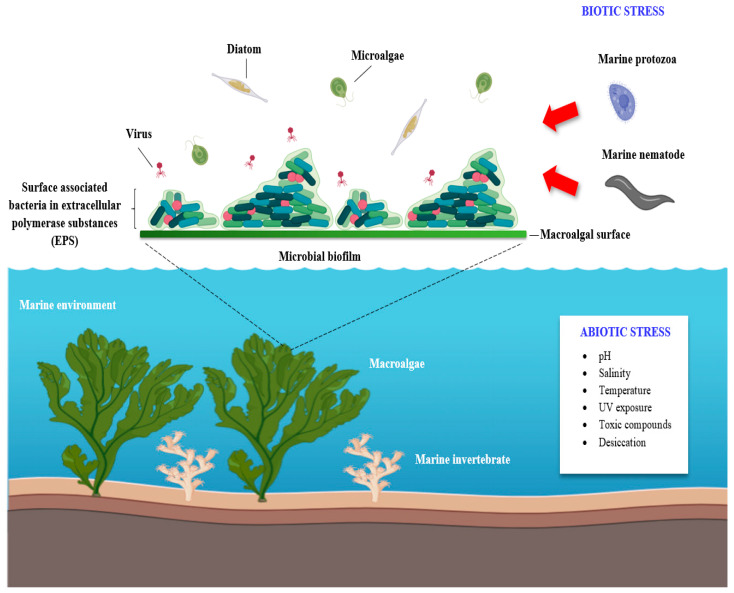
Surface-associated marine bacteria live on the nutrient-rich marine surfaces (for example macroalgae or cnidaria) in the form of biofilms. These marine biofilms are exposed to biotic (intra- and/or inter-species interaction with other microorganisms or predators i.e., protozoa and nematodes) and abiotic physical–chemical stressors. The predator–prey interactions lead to the production of nematicidal metabolites by the surface-associated marine bacteria, while the harsh environmental conditions enhance the chemical, molecular and functional properties of the produced microbial compounds. Image created with BioRender.com.

Marine inhabitants, particularly microorganisms, are continuously exposed to multiple detrimental interactions imposed by competitors and predators [83,84] and different physical–chemical variables such as fluctuating temperature, pH, UV exposure, salinity, toxic compounds and desiccation, particularly in the intertidal zone [85,86,87]. As a survival strategy, some marine bacteria adhere to each other and/or surfaces and are embedded in enclosed matrices to form a biofilm (Figure 1) [88,89].

The continuous development of biofilm on marine surfaces leads to epibiosis, which involves multispecies biofilm formation [90,91]. Macroalgal surfaces, for example, are a hot-spot for colonization by opportunistic epibionts such as algal spores, invertebrate larvae, diatoms, fungi and other bacteria [85,92], mostly due to the accumulation of nutrients and macroalgal exudates composed of organic carbon and nitrogen particles [93,94,95]. Consequently, the competition among marine microorganisms to reserve a space within the biofilm community is tremendously intense and bacterial strains that are equipped with broad-spectrum inhibitory phenotypes are likely to be successful epibiotic colonizers (Figure 1) [83,96].

In addition, predation by heterotrophic protozoa and bacterivorous nematodes represents another biotic stress resulting in major mortality for both planktonic and surface-associated bacteria in the marine habitat (Figure 1) [85,97]. Protozoans, for example *Rhynchomonas nasuta* and *Cafeteria roenbergensis*, are among the most abundant ubiquitous species in the ocean and are the major controllers of the food web in the marine environment through their function as bacterial predators [98,99,100]. Nematodes such as *Pareudiplogaster pararmatus* are among the natural consumers of organic biomass in benthic habitats actively grazing bacterial mats and the biofilms of biotic surfaces [101,102].

The omnipresence of inter- and intra-species interactions supports the evolution of diverse defense strategies by surface-associated marine bacteria. Such defense mechanisms include the production of bioactive compounds showing antibacterial, antifungal, antitumor, antifouling, antiprotozoal and antinematode activities (Table 2) [103,104]. Interestingly, the physical–chemical properties, molecular structure and functional features of those marine microbial compounds are believed to be shaped by the naturally harsh conditions of the marine environment [105]. Moreover, it is speculated that bioactive metabolites originally attributed to marine invertebrates such as sponges, tunicates, bryozoans and molluscs are actually produced by their associated microorganisms [106,107]. For example, the antibiotic peptides andrimid and trisindoline isolated from the sponge *Hyatella* sp. and *Hyrtios altum* are believed to be produced by symbiotic *Vibrio* sp. [108,109]. An antitumor cyclic peptide leucamide A isolated from the sponge *Leucetta microraphis* is closely related to compounds produced by cyanobacterial symbionts [110]. In addition, a commercialized antitumor drug Didemnin B initially isolated from the tunicate *Trididemnum solidum* [111] was recently demonstrated to be produced by symbiotic bacteria *Tistrella mobilis* and *T. bauzanensis* [112,113]. These observations also hold true for numerous antinematode compounds that have been successfully isolated from marine eukaryotes, with production of many now attributed to host-associated microorganisms [51,114].

The potential for marine surface-associated microorganisms to be repositories of unusual gene functions and bioactivities is further corroborated by global biodiversity studies such as the Tara Oceans [69,115] and the Global Ocean Sampling (GOS) expedition [116,117]. These and other studies continue to reveal unprecedented levels of information on microbial diversity, which has subsequently led to investigations on underexplored bacterial diversity in various marine ecosystems. Furthermore, abundant and newly discovered biosynthetic gene clusters encoding rare non-ribosomal peptides (NRPS), polyketides (PKS) and NRPS–PKS hybrids have been discovered from marine biofilm samples, further strengthening the concept of the marine environment as a rich source of newly discovered bioactive compounds [68,69].

**Table 2:** Example of anthelmintic or nematicidal bioactivities isolated from marine bacteria.

Marine Microbial Producer	Compound	Associated Surface/Host	Mode of Action	Responsible Gene(S)	Reference
*Microbulbifer* sp. D250	Violacein	Algae *Delisea pulchra*	Facilitate bacterial accumulation accompanied by tissue damage and apoptosis	*VioA-VioE*	[118]
*Pseudoalteromonas tunicata* D2	Tambjamine	Algae *Ulva australis*	Slow-killing activity by a heat-resistant tambjamine and substantial bacterial colonization in the nematode gut	*TamA-TamT*	[119]
*Pseudoalteromonas tunicata* D2	Unknown	Algae *Ulva australis*	Fast-killing activity by a heat sensitive unknown compound through colonization-independent manner	Unknown	[119]
Uncultured alpha-proteobacterium, JN874385 (strain U95)	Unknown	Algae *Ulva australis*	Undetermined	Possibly NRPS gene	[120]
*Aequorivita* sp.	Unknown	Antarctic marine sediment	Undetermined	Unknown	[121]
* Pseudovibrio * sp. Pv348, 1413, HE818384 (strain D323)	Unknown	Algae *Delisea pulchra*	Undetermined	Unknown	[120]
Heterologous clone jj117 (NCBI Accession number SRX4339430)	Unknown	* Ulva australis * metagenomic library	Undetermined	ATP-grasp protein/alpha-E protein/transglutaminase protein/protease	[122]
*Vibrio atlanticus* strain S-16	Volatile organic compounds (VOC)	Scallop *Argopecten irradians*	Undetermined	Unknown	[123]
*Virgibacillus dokdonensis* MCCC 1A00493	VOC (acetaldehyde, dimethyl disulfide, ethylbenzene and 2-butanone	Polymetallic nodules in the deep sea	Direct contact killing activity, fumigation. Acetaldehyde had a fumigant activity to impede egg hatching	Unknown	[124]
*Pseudoalteromonas rubra*	Unknown	Marine organisms (copepod or fish) or environmental samples	Undetermined	Unknown	[125,126]
*Pseudoalteromonas piscicida*	Unknown	Marine organisms (copepod or fish) or environmental samples	Undetermined	Unknown	[125,126]
*Arthrobacter davidanieli*	Unknown	Marine environmental sample	Undetermined	Unknown	[125,127]
*Pseudoalteromonas luteoviolacea*	Unknown	Marine organisms (copepod or fish) or environmental samples	Undetermined	Unknown	[125,126]
*Photobacterium halotolerans*	Unknown	Marine organisms (copepod or fish) or environmental samples	Undetermined	Unknown	[125,126]

## 4. *Caenorhabditis elegans* as a Model Organism for Antinematode Drug Discovery and Development

Antinematode drug research is impeded by several challenges. These include (i) similarity of biochemical reactions between parasitic nematodes and the infected host, (ii) complex parasite life cycles that involve infections in multiple hosts, (iii) different parasite geographical locations and (iv) rapid development of resistant phenotypes. Therefore, an easily maintained nematode model with the capability for rapid screening of potential antinematode compounds represents a solution to some of these challenges [128,129].

**Figure 2:**
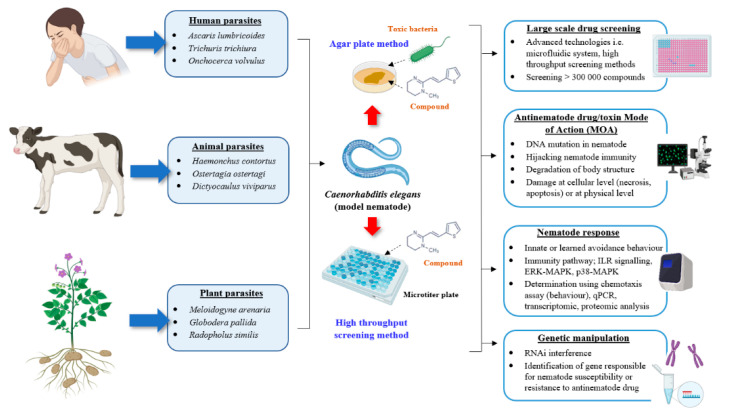
Schematic diagram representing the functional role of *C. elegans* as a model nematode for the development of antinematode drugs. The propagation and handling methods of human, animal or plant-parasitic nematodes in the laboratory are challenging. *C. elegans* can be used as a surrogate nematode for the initial screening against the potential compounds or microorganisms with nematotoxic properties either via the conservative agar plate method or through high throughput screening technologies. *C. elegans*-based research offers several advantages including nematode genetic manipulation, determination of drug MOA, evaluation of the resulting nematode responses and a large-scale initial drug screening due to easy nematode maintenance and propagation in the laboratory. Image created with BioRender.com.

Sydney Brenner and colleagues first introduced the free-living soil nematode *C. elegans* in 1965 as an animal model for research including anti-infective and antinematode drug studies [130,131,132,133,134,135]. Owing to its small size (1–1.5 mm-adult length, 80 µm-diameter), transparency, rapid life cycle (~3 days) and diet based on a simple bacterial culture of *Escherichia coli* OP50, *C. elegans* has emerged as a valuable animal model [134,136,137]. *C. elegans* possess several features which make the organism an efficient and low-cost surrogate organism for antinematode drug discovery. Unlike parasitic nematodes which require vertebrate hosts for reproduction and maintenance [48,138], synchronized *C. elegans* can be easily propagated at the desired life stage on Nematode Growth Media (NGM) ready to be used for antinematode drug studies [139]. The earliest antinematode drug testing using synchronized *C. elegans* individuals was performed by exposing the animals to nematicidal agents incorporated into the agar media [140]. After a few years, the screening protocol evolved rapidly with the development of high throughput screening methods employing micro-fluidic systems or high-content screening (HCS) technologies, allowing for fast and large-scale drug testing (~14,000 to 360,000 of compounds) using *C. elegans* as the model organism (Figure 2) [141,142]. Today, important drug discoveries, such as benzimidazoles [143], ivermectin and its analogues, moxidectin, milbemycin oxime, doramectin, selamectin, abamectin, eprinomectin [144] and the nematicidal activity of crystal protein insecticide Cry5B, Cry21A from *Bacillus thuringiensis* [72,145], can be attributed to the use of *C. elegans* as an effective animal model [48].

*C. elegans* shares many conserved genes and protein functions with parasitic nematodes. Analysis of the intestinal parasite *Strongyloides stercoralis* genetic sequences showed 85% of protein homologs to *C. elegans* genes. The infective stage of *S. stercoralis* (L3i/dauer) also shows an increased proportion of protein homologs to *C. elegans* dauer larvae [146]. Genetic manipulation and RNA interference (RNAi) studies have been widely performed on *C. elegans* to provide a better understanding of the nematode response against the nematicidal drugs at the molecular level (Figure 2). Quantitative polymerase chain reaction (qPCR) and “omic” technologies, e.g., transcriptomic profiling and proteomics, are also being used to provide a global snapshot of the molecular response of *C. elegans* to drug exposure [147,148]. Given the conserved gene homologs and protein function among the members of phylum Nematoda, these studies provide insight into the possible mechanisms used by parasitic nematodes against similar drug exposure [48].

The majority of the current drugs used to treat parasitic nematode infections target proteins that regulate neuromuscular activity including neurotransmitter receptors and ion channels [48]. Utilization of *C. elegans* as a model organism confers a better understanding of the mode of action (MOA) of potential nematicidal drugs (Figure 2). Studies have shown that *C. elegans*’ neuromuscular system, its major neurotransmitters GABA (4-aminobutyric acid) and glutamate and its enzyme choline acetyltransferase, responsible for the synthesis of the neurotransmitter acetylcholine, display strong similarities to parasitic roundworms *Ascaris suum* and *Ascaris lumbricoides* [48,149,150,151,152]. Therefore, exposure of *C. elegans* to antinematode compounds which target the neurotransmitter receptor and ion channels has enabled the discovery of the target binding molecule and the resulting toxicity to parasitic nematodes [153]. For example, observations of body muscle contraction and spastic paralysis in *C. elegans* exposed to levamisole, led Lewis and colleagues [154] to determine that binding to the muscle acetylcholine receptors was key to its activity. The initiation of amino-acetonitrile derivative (AAD) toxic activity against nematodes by binding to a nicotinic acetylcholine receptor was also revealed via forwards genetic screening using *C. elegans* mutants [155]. More recently, the MOA of paraherquamide, a broad-spectrum nicotinic nematicidal alkaloid isolated from *Penicillium paraherquei* [156], and antinematode plant-derived compounds [157] were also elucidated using *C. elegans* [157,158,159]. While using *C. elegans* to identify specific targets is promising, assessing side effects related to these newly discovered compounds will be important considering many of the neurotransmitter targets will also be present in the host organism [48]. Nevertheless, in the event that novel drugs negatively impact non-target organisms, derivatives may be developed with reduced side effects [160,161].

## 5. Conclusions

The increasing prevalence of parasitic nematode infection and the emergence of antinematode drug resistance represent critical global issues, impacting human wellbeing and economic development. Moreover, increasing temperatures and changing weather patterns, moisture and rainfall as a result of global climate change may also escalate the prevalence of parasitic nematode diseases worldwide [40,41,42,43,44]. Although for decades many anthelmintic/nematicidal drugs have been successfully derived from terrestrial microorganisms (see examples in Table 1), the paucity of new antinematode drug classes and the challenges now presented with drug resistance underline the importance of discovering new antinematode bioactive metabolites from natural sources [4,50]. This review highlighed the potential of marine epiphytic bacteria as a new platform for novel antinematode drug development. Marine epiphytic bacteria are highly diverse, harboring unique genes expressing newly discovered bioactive metabolites with commercially or pharmaceutically relevant biological potentials including antinematode activities [118,119,120]. Owing to the extraordinary molecular structure and physical/chemical properties, marine bioactive compounds are regarded as “blue gold from the ocean” and are believed to be a promising source for future novel antinematode drugs. The ability to uncover these novel marine-derived antinematode drugs will be dependent on the successful implementation of innovative cultures, culture-independent techniques and high-throughput bioassays, for which the model nematode *C. elegans* is well suited.
